# Mitigation of B_1_^+^ inhomogeneity for ultra-high-field magnetic resonance imaging: hybrid mode shaping with auxiliary EM potential

**DOI:** 10.1038/s41598-020-68651-6

**Published:** 2020-07-16

**Authors:** Minkyu Park, Hansol Noh, Namkyoo Park

**Affiliations:** Photonic Systems Laboratory, Department of Electrical and Computer Engineering, Seoul National University, Seoul, 08826 Korea

**Keywords:** Applied optics, Applied physics, Magnetic resonance imaging

## Abstract

The notion of mode shaping based on evanescent coupling has been successfully applied in various fields of optics, such as in the dispersion engineering of optical waveguides. Here, we show that the same concept provides an opportunity for the seemingly different field of ultra-high-field MRI, addressing transmit RF magnetic field (B_1_^+^) inhomogeneity. In this work, treating the human phantom as a resonator, we employ an evanescently coupled high-index cladding layer to study the effects of the auxiliary potential on shaping the B_1_^+^ field distribution inside the phantom. Controlling the strength and coupling of the auxiliary potential ultimately determining the hybridized mode, we successfully demonstrate the global 2D homogenization of axial B_1_^+^ for a simplified cylindrical phantom and for a more realistic phantom of spheroidal geometry. The mode-shaping potentials with a magnetic permeability or material loss are also tested to offer additional degrees of freedom in the selection of materials as well as in the manipulation of the B_1_^+^ distribution, opening up the possibility of B_1_^+^ homogenization for 3D MRI scanning.

## Introduction

Modal shaping with evanescent coupling has been employed as a proven technique in various applications of optics^1–4^. For example, double-clad W-type fibers use a higher-index outer cladding layer in combination with a lower-index inner cladding layer to tailor the modal confinement and dispersion properties^5–7^, which is not achievable from simple core-clad structures. A problem that is similar in nature also exists in different applications and carrier frequencies of magnetic resonance imaging (MRI) under the terminology of RF shimming or B_1_^+^ inhomogeneity mitigation. In the MRI system, the RF electromagnetic field B_1_^+^ is generated by the RF coil, tuned around the Larmor frequency *ω*_*L*_, which is set by and scales with the strength of the DC bias magnetic field (B_0_). While the most commonly used MRI systems use a B_0_ of 3 T (Tesla), the latest MRI devices employ a higher magnetic field, such as 7 T, to enhance the signal-to-noise ratio (SNR), spatial and temporal resolutions, and contrast^8–11^.

Nonetheless, with the increase in bias B_0_, the corresponding Larmor wavelength *λ*_*L*_ for the B_1_^+^ RF field also decreases, causing B_1_^+^ inhomogeneity problems in MRI. In ultra-high-field (UHF) MRI over B_0_ = 7 T, with the high permittivity of body tissue (*ε* ~ 78 at *ω*_*L*_ = 300 MHz), the Larmor wavelength of the B_1_^+^ field in the body (*λ*_L_ ~ 11 cm) becomes comparable to or smaller than the size of the human body. This high permittivity body surrounded by air of much lower permittivity *ε* = 1 then constitutes an electromagnetic resonator operating at the Larmor RF frequency under the excitation of the MRI RF field B_1_^+^. This body resonator then inherently presents the typical field pattern of a few-mode resonator (e.g., bright in the central region, dark in the periphery)^12,13^, and inhomogeneity in the B_1_^+^ field in MRI arises, which is detrimental to homogeneous retrieval of the intensity, SNR, and contrast in MRI applications^14–17^.

Common approaches for mitigating or shimming this B_1_^+^ inhomogeneity are to use the capacity to alter the B_1_^+^ distribution with high-permittivity materials (HPM). Dielectric pads filled with water (*ε* ~ 78 at 300 MHz) placed in contact with the head have been used to improve the sensitivity of the signal in the peripheral region of the ROI (region of interest), especially in proximity to the high permittivity pads^18^. Pads of mixtures of metal titanate and water have been employed to increase the permittivity of the pads so that they can be used in similar strategies^19–24^. Structures with metallic inclusions, such as metasurfaces and hybridized meta atoms (HMAs), have also been reported to manipulate field profiles at subwavelength scales^25,26^. However, while successful in controlling the field distribution, most of the past efforts utilizing the local enhancement of B_1_^+^ in the *vicinity* of the pad structures, especially those in contact with the body, often resulted in deterioration of the *global* B_1_^+^ homogeneity over the ROI.

In this paper, we apply the notion of optical mode shaping to UHF MRI systems and successfully achieve global 2D homogenization of the axial B_1_^+^ field. We treat the MRI system, composed of the body and an RF B_1_^+^ coil, as a waveguide operating at the Larmor wavelength and then employ a high permittivity cladding layer (auxiliary electromagnetic potential well) to tailor the mode shape inside the body through controlled evanescent mode coupling. For this, we control three key parameters of the RF B_1_^+^ waveguide: depth of the pad potential (cladding layer permittivity), width of the pad potential, and width of the potential barrier (air gap) between the body potential and the pad potential. From the results of a numerical analysis employing the simplified phantom of the brain, we then derive an optimal layout of the geometry and material parameters of the pad, which can be applied to the MRI systems in 2D scanning operations. Finally, we also examine the cases of pad potential with magnetic permeability or material loss to reveal additional degrees of freedom in the manipulation of the B_1_^+^ distribution, which can be used as a route for 3D MRI shimming.

## Results

### Concept of hybrid mode shaping with auxiliary EM potential

**Figure 1:**
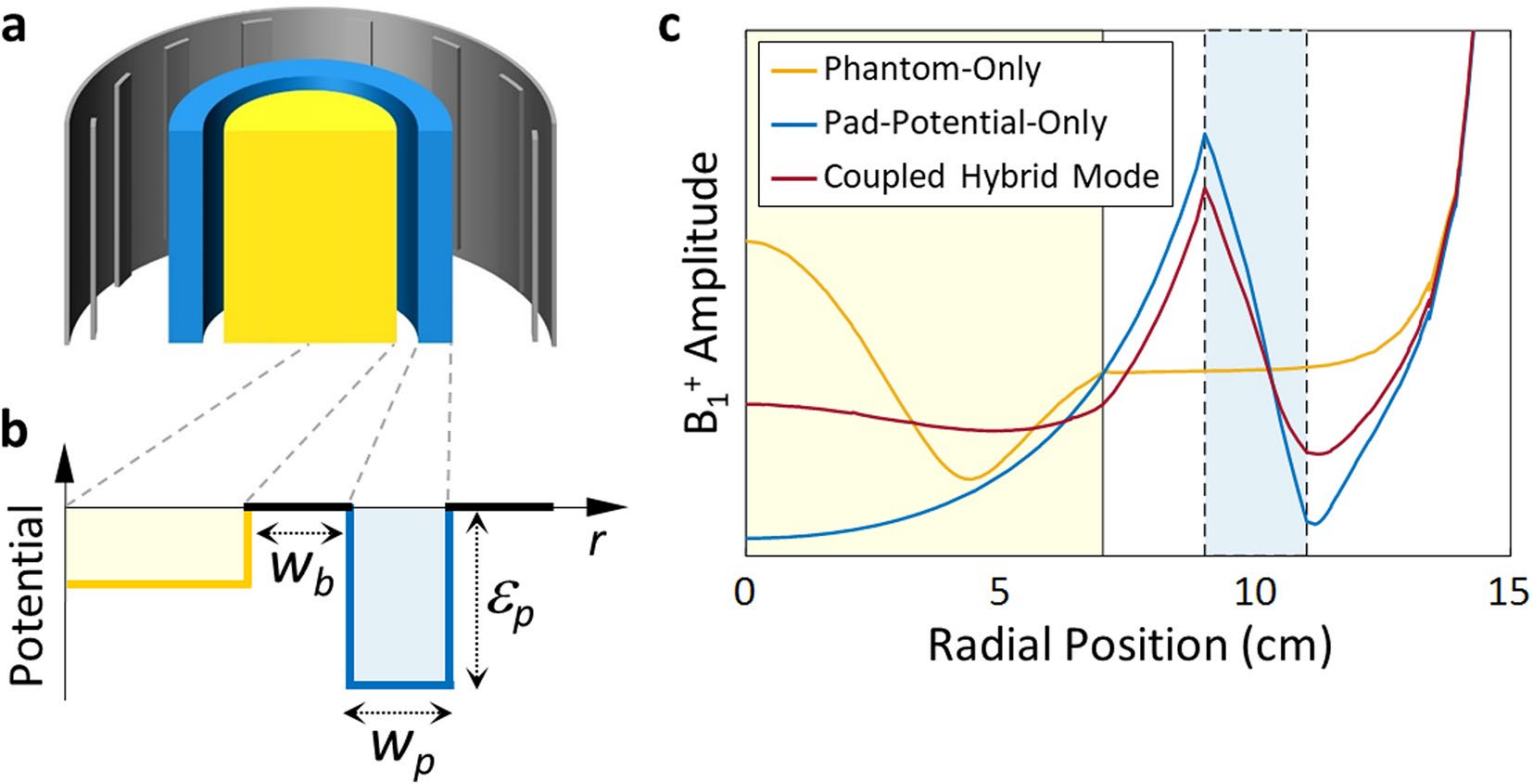
The concept of evanescent mode coupling for MRI B_1_^+^ shimming with the pad potential. (**a**) Schematic of the proposed MRI B_1_^+^ shimming system: RF coil (gray), phantom (yellow), and cylindrical pad (blue). (**b**) The corresponding permittivity potential layout along the radial direction: phantom (yellow), air-gap barrier (black), and pad (blue line). (**c**) Plot of the B_1_^+^ field amplitude along the radial direction at the center of the axial plane (z = 0): phantom-only (yellow curve), pad-potential-only (blue curve), and both the phantom and pad potentials (red curve). The yellow- and blue-shaded region each corresponds to the brain phantom and pad.

Considering the structure of MRI and the corresponding symmetries of the RF B_1_^+^ and DC B_0_ fields, it is reasonable to approach the present solution as an electromagnetic boundary problem having dominant translational symmetry in the sagittal direction, with finite length. Since the uniformities of the B_1_^+^ field distribution in the radial and sagittal directions have a trade-off relation in a cylindrical phantom at a given Larmor frequency^27^, we focus here on axial 2D MRI scanning, which is widely used in clinical applications. To start, we first simplify the MRI system to maintain the *z*-translational symmetry by assuming a *cylindrical* phantom and a *cylindrical* pad to minimize the mode mixing between the radial (*r*), sagittal (*z*), and azimuthal (*θ*) directions (Fig. 1a). In this setting, at a fixed sagittal (*z*-axis) height of the B_1_^+^ RF coil, phantom, and pad, the shaping of the mode is then determined by three control parameters: the depth of the auxiliary pad potential *d*_*p*_, the width of the pad potential *w*_*p*_, and the width of the potential barrier (air gap) *w*_*b*_ (Fig. 1b). We further note that in view of evanescent coupling, it is clear that the control of *d*_*p*_ and *w*_*p*_ will govern the amplitude and width of the B_1_^+^ field in the pad, while the control of the potential barrier *w*_*b*_ will determine the evanescent coupling and mode hybridization between the phantom and pad modes, achieving the resultant mode shaping in the target ROI.

To assess the effect of mode hybridization from the proposed evanescent mode coupling, full-wave electromagnetic numerical analyses are carried out by using an FEM-based simulation tool (COMSOL Multiphysics). As the source of B_1_^+^ field RF excitation, we assume an idealized coil consisting of 16 surface current sources (rectangular, 1 cm × 18 cm) equally spaced on a cylinder (radius 15 cm, height 18 cm). The currents on each source are sinusoidally driven with the same magnitude at a Larmor frequency of 300 MHz (corresponding to B_0_ = 7 T) and are sequentially phase-shifted by 360°/16 = 22.5°. It is noted that this sequential phase shift provides a homogeneous distribution of circularly polarized fields (i.e., B_1_^+^) with a coefficient of variation of less than 6% in the phantom loading region. The coil is surrounded by a cylindrical surface of the perfect electric conductor (PEC) with a radius of 17 cm and a height of 18 cm, modeling the shield of the coil for the MRI systems. To start, we also focus on the imaging of the head and employ a simplified head phantom, assuming a lossy dielectric cylinder (height of 18 cm, radius of 7 cm, relative permittivity of 74.2, conductivity of 0.87 S/m) made of an agar–agar gel^26^. For the dielectric pad encircling the phantom, we also assume a high-permittivity material and cylindrical geometry, to emphasize, not in contact but evanescently coupled to the head phantom through the potential barrier of the air gap. These shielded coil, cylindrical pad, and phantom constitute a coaxial structure, as shown in Fig. 1a,b, with air gaps in between.

Figure 1c shows the excited modes without or with the presence of pad potential: the yellow and the blue curve correspond to the B_1_^+^ field amplitude with the phantom only and the pad only, respectively, while the red curve shows that of the two potentials together, forming a hybridized mode exhibiting a flattened mode profile in the ROI of the head (at *z* = 0). Depending on the relative strength of the phantom and pad potentials with gaps in between, each potential dominates the overall mode profile in the MRI system, perturbed by the other potential. It is also critical to note that for a pad potential of sufficiently high permittivity, higher than that of the phantom, the behavior of the coupled mode is dominated by the pad potential and not greatly perturbed by the geometry or permittivity distribution of the phantom.

### Optimization process

While some clinical imaging prefer B_1_^+^ profiles brighter in the central region of the ROI, a flattened mode profile is preferred in conventional, 2D MRI scanning. Here, focusing on the 2D homogenization of B_1_^+^ over the transverse plane, in terms of the control parameter set (*d*_*p*_, *w*_*p*_, *w*_*b*_), we look into the following figures of merit (FOMs): (1) B_1_^+^ maximum-to-minimum ratio (MmR) over the axial plane (*r*, *θ*) signifying the degree of 2D flattening; (2) the position of the B_1_^+^ node in the radial direction (*r*_*node*_) representing the redistribution of the B_1_^+^ fields; and (3) the contour of B_1_^+^ at the plane (*r*, *z*) showing homogenization over the *z* direction.

**Figure 2:**
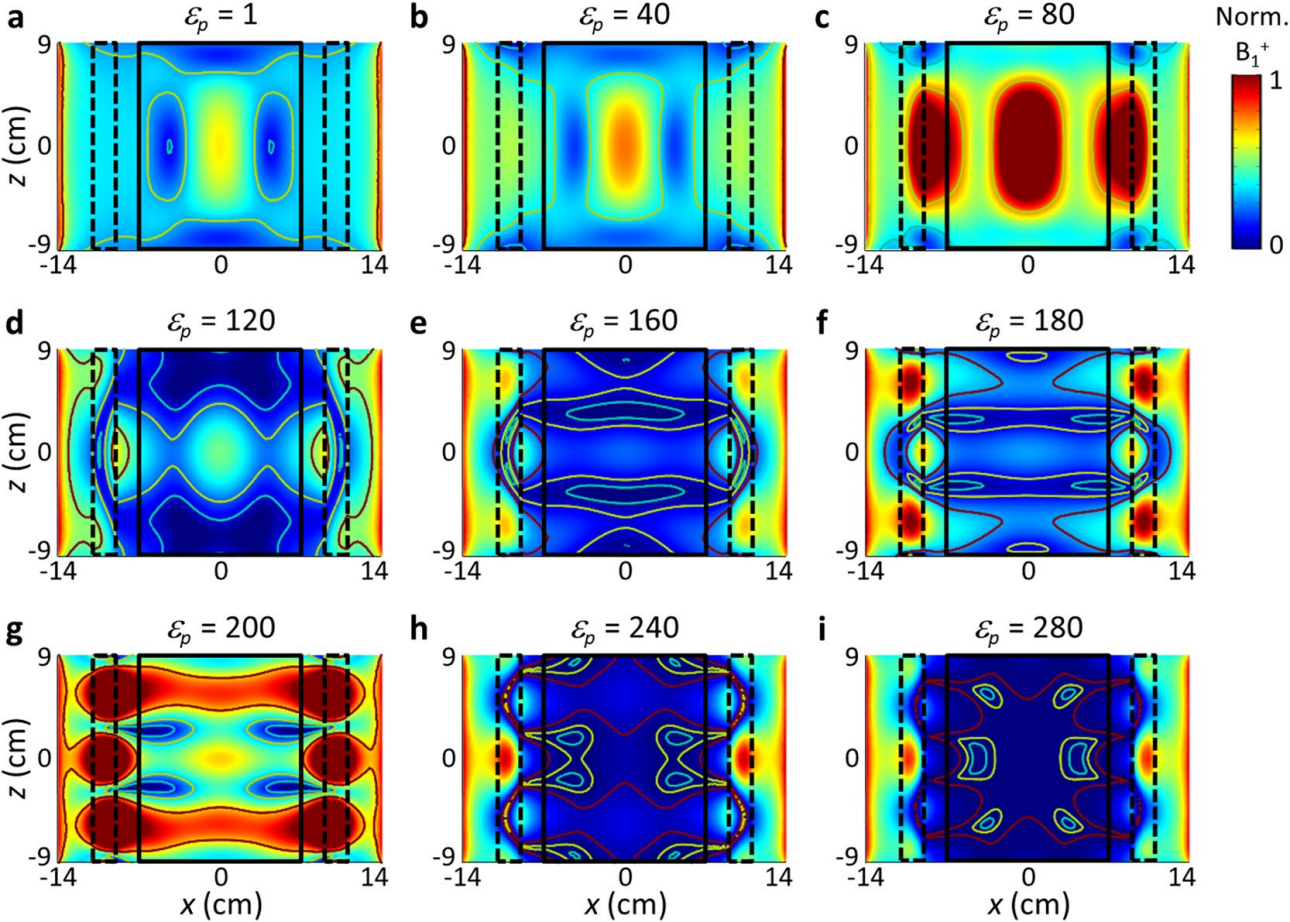
Effects of the pad permittivity *ε*_*p*_ (pad potential depth) on the mode shaping. Normalized field amplitude in the sagittal plane (*θ* = 0). Contour lines represent the B_1_^+^/B_1_^+^_*center*_ values of 1 (red), 1/2 (yellow), and 1/4 (blue). The region of interest, i.e., the cylinder phantom, is marked with a black rectangle. The pad is marked by the black-dashed rectangles. The width of the air-gap potential barrier *w*_*b*_ is set at 2 cm. The width of the pad potential well *w*_*p*_ is set at = 2 cm.

Figure 2 shows the effect of the pad potential-well depth *d*_*p*_ on the mode profile of the phantom. When the pad potential is shallower than that of the phantom (*ε*_*p*_ < 80) (Fig. 2a,b), the effect of the pad on the mode shaping is not significant, with the deeper and wider potential of the phantom dominating the coupled system. On the other hand, with a pad permittivity *ε*_*p*_ > 200 (Fig. 2h,i), the B_1_^+^ field is strongly confined in the pad, inhibiting its coupling to the phantom. At fixed *w*_*b*_ = 2 cm and *w*_*p*_ = 2 cm, with *ε*_*p*_ = 180, the lowest axial MmR of 1.2 is obtained, far lower than the MmR of 4.3 for the phantom-only case (*ε*_*p*_ = 1).

**Figure 3:**
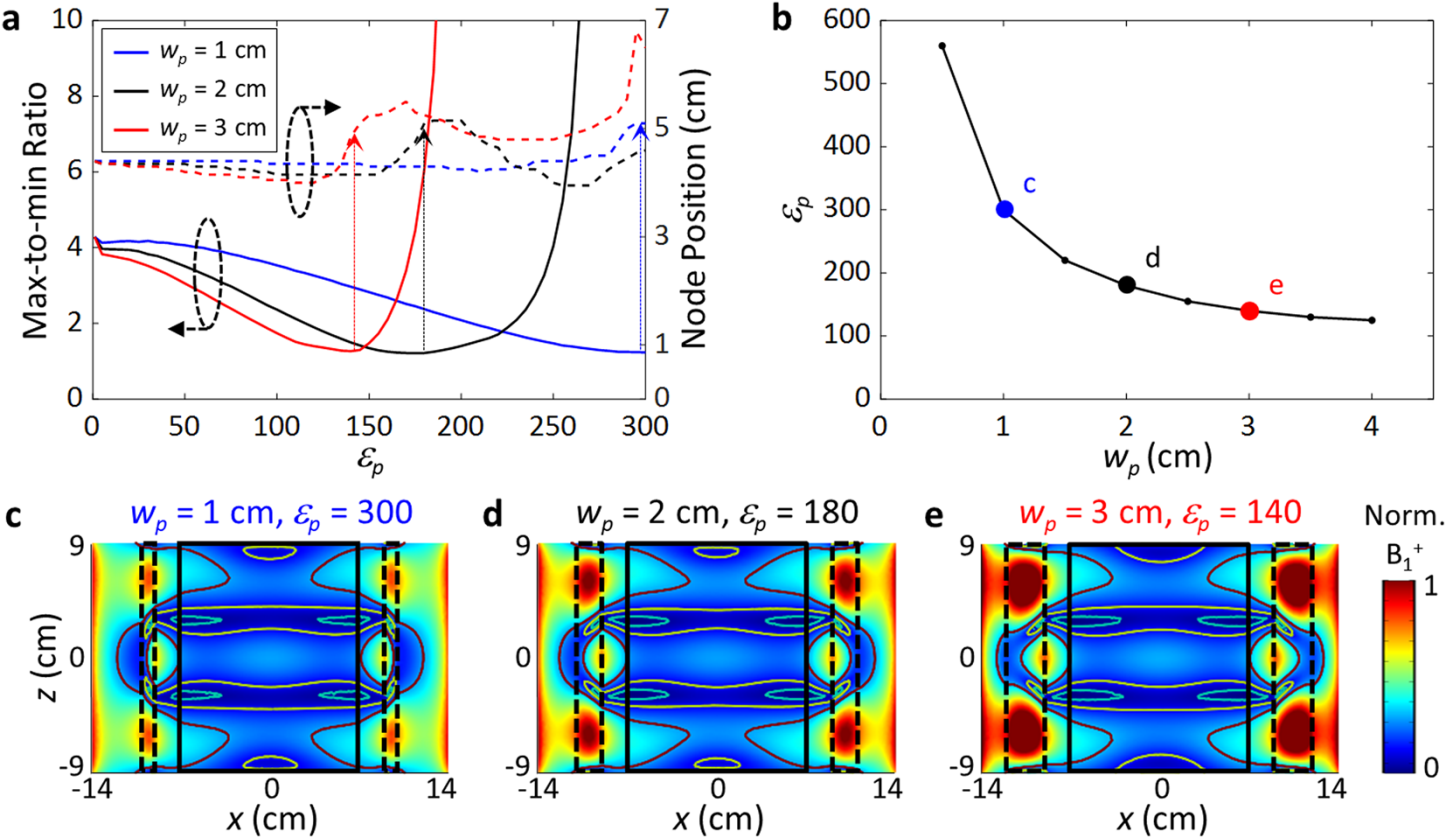
Effects of the pad potential width *w*_*p*_ on the mode shaping. (**a**) Max/min ratio (left axis, solid curves) and node position (right axis, dotted curves) obtained for various *w*_*p*_ (1–3 cm), at the central plane of the ROI (*z* = 0). (**b**) Relation between *w*_*p*_ and *ε*_*p*_, obtained for the minimum MmR = 1.2. (**c-e**) Mode profile in the sagittal plane (*θ* = 0). Potential barrier width *w*_*b*_ = 2 cm.

Figure 3 shows the effect of the pad width *w*_*p*_ at various *ε*_*p*_. As expected, the depth and width of the potential operate in a complementary manner providing almost the same pattern of modes and MmR values, e.g., for sets (*ε*_*p*_, *w*_*p*_) = (140, 3 cm), (180, 2 cm), and (300, 1 cm), implying an extra degree of freedom in the selection of pad materials (Fig. 3b). It is also noted that the lowest MmR is achieved when the node positions are farthest from the center, signifying the redistribution of the B_1_^+^ fields in the radial direction for the shimming.

**Figure 4:**
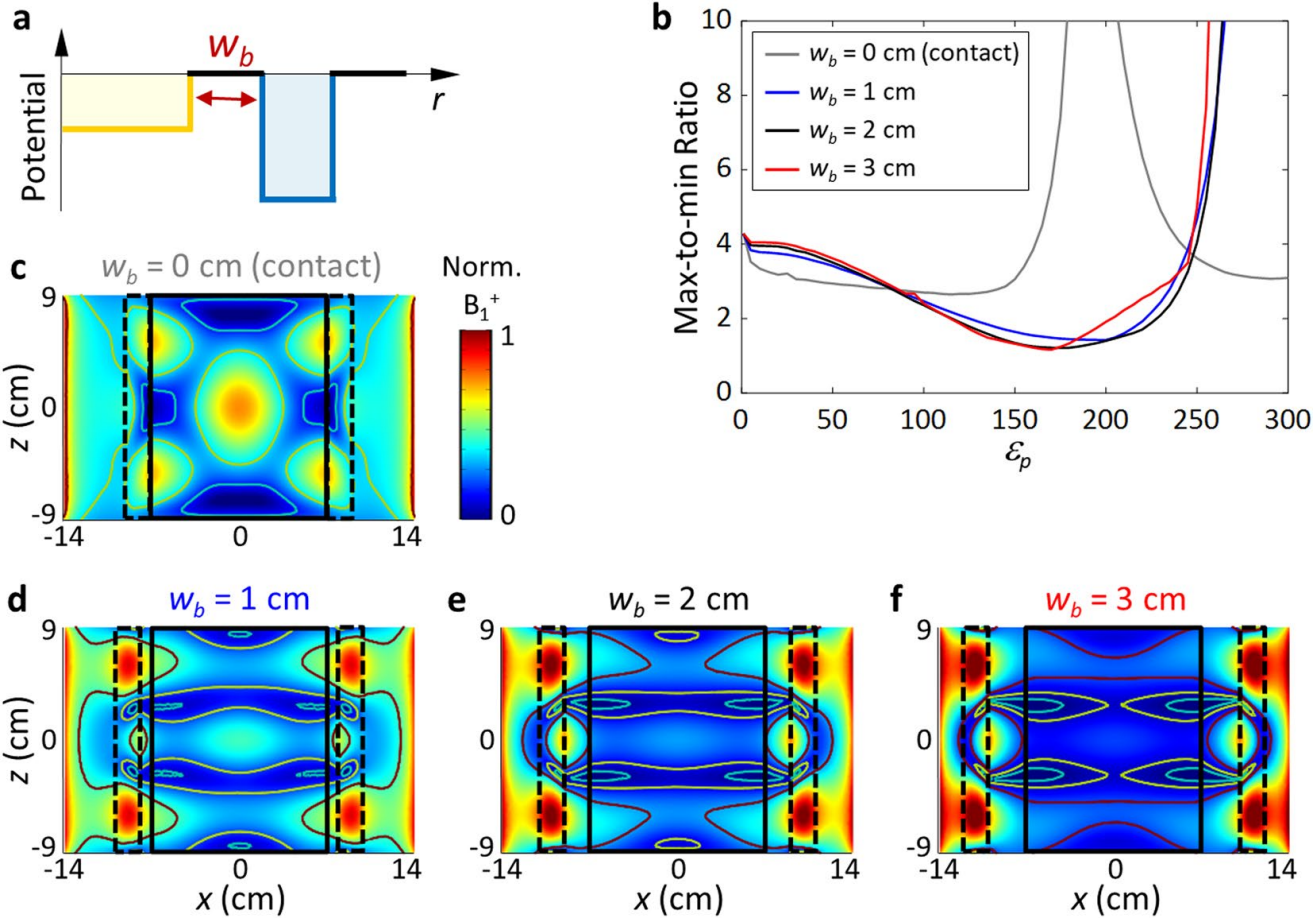
Effects of the air-gap potential barrier width *w*_*b*_ on the mode. (**a**) Potential distribution in the radial direction. (**b**) Max/min ratio obtained for various *w*_*b*_ values at the central plane of the system (z = 0). (**c–f**) Mode profile in the sagittal plane (*θ* = 0). *ε*_*p*_ = 180, and *w*_*p*_ = 2 cm.

Figure 4 shows the effects of the air-gap barrier width *w*_*b*_ on the mode shaping. It is noted that *w*_*b*_ = 0 refers to a pad in contact with the phantom, and *w*_*b*_ > 0 corresponds to the evanescently coupled potential case. By controlling *w*_*b*_, it is possible to adjust the strength of the evanescent coupling from the high-permittivity pad while retaining the general shape of the pad mode. From this added degree of freedom, we find that a nonzero *w*_*b*_ consistently provides much better homogeneity than the contact pad (*w*_*b*_ = 0), which has been demonstrated previously^18,20,21,28,29^. An MmR value of 1.2 is obtained, far lower than that in the case of the contact pad (MmR >  ~ 2.6). Furthermore, we note that the stability of our solution against a range of *w*_*b*_ values implies the robustness of our evanescent mode-shaping solution to the shape or displacement of the used phantom. In the optimization process, only three factors (effectively two) need to be considered for homogenization, which is less complex than the pad optimization scheme in other studies^30,31^.

### Effect of the phantom geometry and other material parameters of the pad

**Figure 5:**
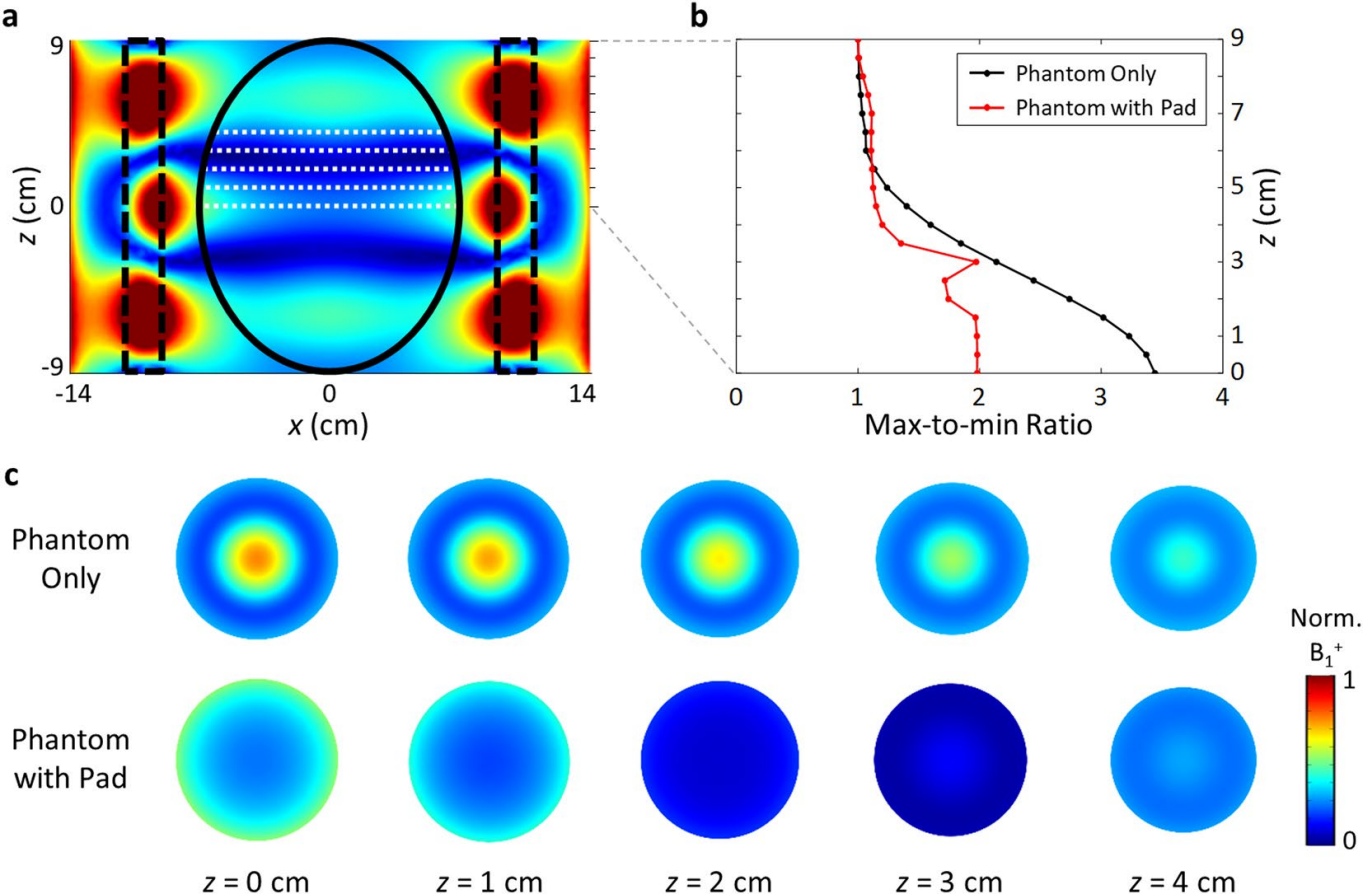
B_1_^+^ homogenization tested with a spheroidal phantom (long axis = 9 cm, short axis = 7 cm). For the pad, *ε*_*p*_ = 188, *w*_*b*_ = 2 cm, and *w*_*p*_ = 2 cm. (**a**) Mode profile in the sagittal view. (**b**) Max/min ratio at different axial slices (*z*-positions). (**c**) Axial images along the *z*-direction.

By replacing the cylindrical phantom with a more realistic one of prolate spheroid geometry (semiaxis of 7 cm along the *x*-direction, 7 cm along the *y*-direction, and 9 cm along the *z-*direction), we continue the optimization of the pad geometry for 2D scanning MRI. It is found that the B_1_^+^ mode profile is not critically sensitive to the geometry of the tested phantoms unless the size of a phantom changes dramatically. Especially when the pad mode dominates the overall system with a high pad-permittivity value, the geometry of the phantom is perturbative only to the overall system behavior. Considering that the homogeneity of the B_1_^+^ field over a sliced plane affects the performance of the 2D scanning MRI system, we focus on the in-slice B_1_^+^ uniformities of the system. Figure 5 shows MmR values and mode profiles along different *z-*cut planes without and with the mode-shaping pad. It is noted that the two nodes of B_1_^+^ at *z* ~  + /− 2 cm are the signatures of the Fabry–Perot resonance modes of the high-permittivity pad along the *z-*direction. Overall, the max/min ratio of B_1_^+^ in a transverse plane is kept less than 2 throughout the phantom. Specifically, with the cylindrical pad, a large reduction (max of 57%) of the MmR is observed in the central region (*z* = − 4 to 4 cm) of the spheroid phantom, where B_1_^+^ field focusing is usually observed without the application of an evanescently coupled pad.

**Figure 6:**
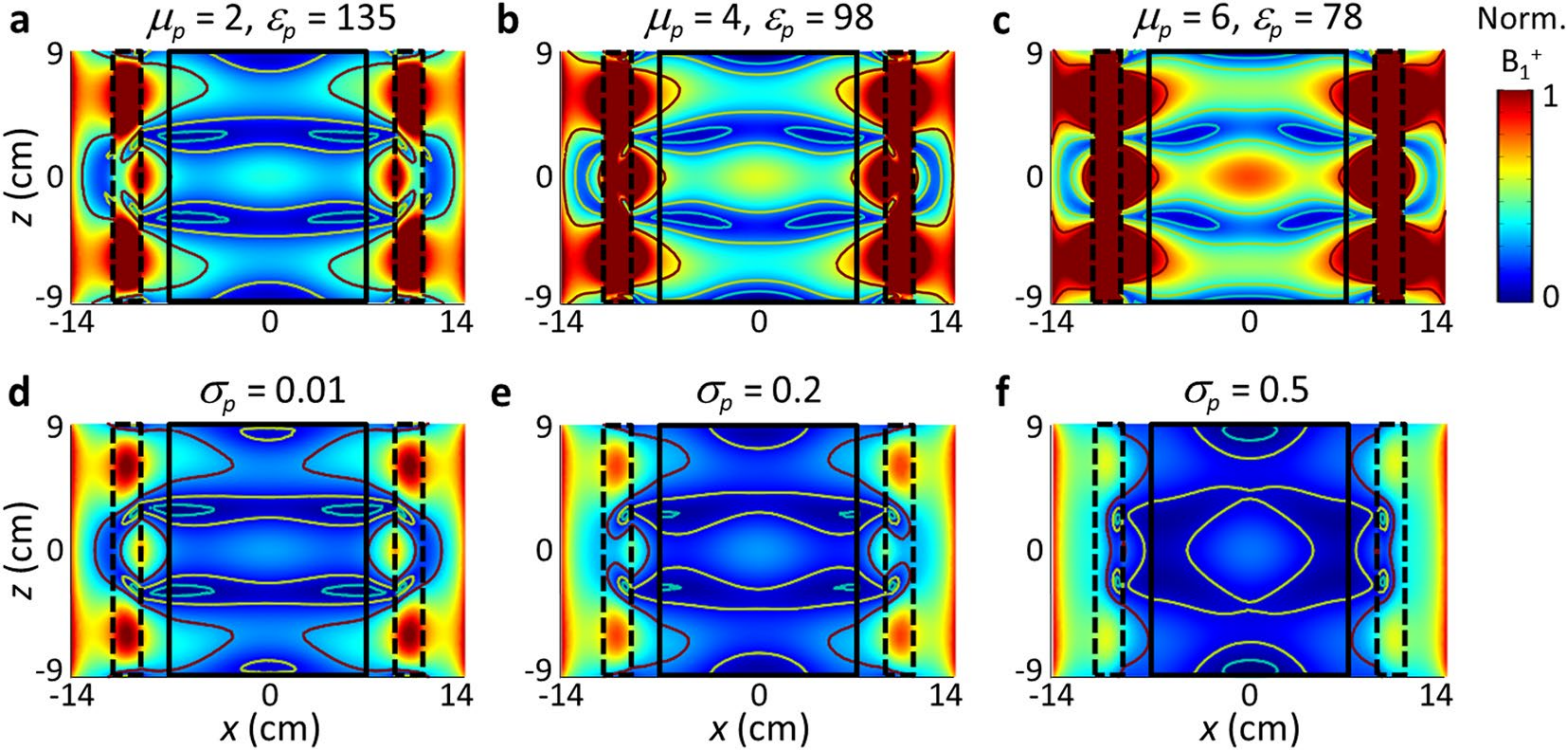
Effect of the magnetic permeability and the loss of the pad. (**a-c**) Mode profile for a magnetic pad of *μ*_*p*_ = 2, 4, 6. (**d-f**) Effects of pad loss (*σ*_*p*_ = 0.01, 0.2, 0.5) on the mode shaping. *ε*_*p*_ = 180. For all cases, *w*_*b*_ = 2 cm, and *w*_*p*_ = 2 cm.

In view of the refractive index, the magnetic permeability of the pad should work as an equivalent potential for the B_1_^+^ RF field in the same way as the electric permittivity discussed earlier. In Fig. 6a–c, as an additional degree of freedom in the design of the B_1_^+^ shimming pad, we present cases of magnetic pads (*μ*_*p*_ = 2, *ε*_*p*_ = 135), (*μ*_*p*_ = 4, *ε*_*p*_ = 98), and (*μ*_*p*_ = 6, *ε*_*p*_ = 78), such as those based on metamaterials, providing a similar B_1_^+^ RF field pattern to that of nonmagnetic pads (*μ*_*p*_ = 1, *ε*_*p*_ = 180). In Fig. 6d–f, we also show the effect of pad loss (or the imaginary part of the permittivity, *σ*_*p*_) on the mode shaping. For a non-negligible damping of *σ*_*p*_ > 0.01, the *z*-directional Fabry–Perot resonance in the pad is suppressed, decreasing the contrast or inhomogeneity of the B_1_^+^ field in the *z*-direction. Together, these results offer strategies for the selection of materials, and the pad-based 3D shimming of the B_1_^+^ RF field.

## Discussion

To summarize, we propose the concept of B_1_^+^ field mode shaping in the notion of evanescent coupling of an auxiliary potential to address the issue of transmit RF (B_1_^+^) inhomogeneity in UHF MRI. Specifically, treating the head-air-HPM (high-permittivity materials) MRI system as an RF waveguide excited by an external electromagnetic source, we systematically optimize the material and geometrical parameters of the HPM and evanescent coupling for B_1_^+^ shimming and prove its validity against the conventional contact-pad structure. It is revealed that the mode shape and homogeneity inside the phantom are determined by two key factors: the mode shape inside the pad potential and the hybridization strength between the phantom and the pad potentials. The mode shape inside the pad potential is again dictated by the electric permittivity of the pad (*ε*_*p*_), the width of the pad (*w*_*p*_), the magnetic permeability of the pad (*μ*_*p*_), and the loss of the pad (*σ*_*p*_). The hybridization strength is controlled by the width of the air potential (*w*_*b*_), working as a potential barrier between the phantom and pad potentials. With the strong boundary condition derived from the high permittivity of the pad (*ε*_*pad*_ >  > *ε*_*phantom*_), we achieve a robust, global homogenization of axial B_1_^+^ throughout the ROI with negligible dependence on the geometry of the phantom. With a simplified phantom of spheroidal geometry, the max-to-min ratio of B_1_^+^ was kept < 2 over the whole region, being applicable to 2D MRI scanning. With the use of additional parameters, such as a noncylindrical pad geometry, an anisotropic HPM material and a metasurface HPM, further extension of our proposal should be possible for higher-level B_1_^+^ mode control in UHF MRI.
